# Comparison of nab-paclitaxel, paclitaxel, and oxaliplatin-induced peripheral neuro-pathy: a cross-sectional cohort study

**DOI:** 10.2340/1651-226X.2025.42935

**Published:** 2025-04-15

**Authors:** Terry Trinh, Kimberley Au, Arun V. Krishnan, Hannah C. Timmins, Tiffany Li, Peter Grimison, David Goldstein, Susanna B. Park

**Affiliations:** a School of Clinical Medicine, University of New South Wales, Sydney, Australia; b Faculty of Medicine and Health, Brain and Mind Centre and School of Medical Sciences, Faculty of Medicine and Health, University of Sydney, Sydney, Australia; c Chris O’Brien Lifehouse, Camperdown, Australia; d Department of Medical Oncology, Prince of Wales Hospital, Randwick, Australia

**Keywords:** chemotherapy-induced peripheral neuropathy, toxicity, cohort study, cancer survivorship

## Abstract

**Background and purpose:**

There remains limited evidence regarding the relative neurotoxic potential of nab-paclitaxel long-term. This cross-sectional matched cohort study aimed to compare the severity and natural history of chemotherapy-induced peripheral neuropathy (CIPN) between patients treated with nab-paclitaxel and patients treated with oxaliplatin or paclitaxel using a suite of clinical, patient reported, and neurophysiological assessments.

**Patients and methods:**

CIPN assessments included the total neuropathy score (TNSc), grooved pegboard assessment, sensory assessments (grating orientation task and Von-Frey assessment), patient-reported outcome measures, and neurophysiological studies. The matched cohort included 72 patients (24 nab-paclitaxel, 24 paclitaxel, and 24 oxaliplatin); each stratified into two groups by time post-treatment, 0–4 months, and > 6 months.

**Results:**

Chronic neuropathy was reported by 71% of nab-paclitaxel-treated patients, most prominently numbness in the lower limbs. Longer time post-treatment was associated with significantly better TNSc scores (*p* = 0.044). Neuropathy severity was similar for nab-paclitaxel and paclitaxel. Neuropathy severity was worse for oxaliplatin compared to nab-paclitaxel, as measured by sensory function (*p* < 0.05) and sural amplitude (*p* = 0.003), but similar for patient-reported and neurological-graded assessments.

**Interpretation:**

These findings underscore the importance of inclusion of a range of multimodal CIPN assessments to characterize relative burden of toxicity between chemotherapy agents. CIPN severity was similar between nab-paclitaxel and paclitaxel cohorts, but more severe in oxaliplatin-treated patients.

## Introduction

Chemotherapy-induced peripheral neuropathy (CIPN) is a common side effect of chemotherapies, including platinum compounds, taxanes, vinca alkaloids, and bortezomib [1]. CIPN predominantly affects sensory nerves and causes paraesthesia, numbness, and discomfort in a ‘glove and stocking’ distribution [1]. These symptoms lead to functional disability, affecting fine motor function, walking, and balance. Accordingly, CIPN is a common cause of dose reduction and early treatment cessation [2].

CIPN natural history has been comprehensively profiled for chemotherapies, including paclitaxel and oxaliplatin [3, 4]. However, there has been limited evidence regarding the natural history of nab-paclitaxel-induced CIPN [5]. Nab-paclitaxel is an albumin-bound nanoparticle formulation of paclitaxel that is indicated as a treatment for metastatic pancreatic and breast cancers. Meta-analyses have suggested higher prevalence of neurotoxicity with nab-paclitaxel compared to paclitaxel [6, 7]; however, there remains a lack of data examining CIPN with multimodal assessment tools. Although there remains no gold standard assessment tool for CIPN, patient reported outcome measures, and clinical assessment tools and neurological grading scales provide complementary but distinct information about CIPN [1, 8].

Accordingly, this study aimed to compare the CIPN phenotype of nab-paclitaxel-treated patients to paclitaxel and oxaliplatin-treated patients using patient-reported outcomes, functional assessments, and neurophysiological techniques.

## Methods

Nab-paclitaxel, paclitaxel, or oxaliplatin-treated patients were recruited to an observational, cross-sectional cohort study from Australian oncology treatment centers between 2014 and 2020. This study was conducted with the ethical approval from South Eastern Sydney Local Health District and Sydney Local Health District (RPAH zone) Human Research Ethics Committees. All patients provided written informed consent in accordance with the Declaration of Helsinki.

Patients were assessed at or after completion of neurotoxic chemotherapy. Nab-paclitaxel-treated participants who received additional neurotoxic chemotherapy during the follow-up period were excluded. Participants were compared to 1:1 cohorts of oxaliplatin or paclitaxel-treated patients who were matched on age, diabetes status, body mass index, and time post-treatment to nab-paclitaxel-treated patients.

### Assessment tools

CIPN was assessed using multimodal tools incorporating neurological examination, sensory and functional assessments, and patient-reported outcomes. Overall neuropathy burden was graded using the National Cancer Institute Common Terminology for Adverse Events Sensory neuropathy subscale (version 4) to categorize CIPN into grades from 0 (no neuropathy) to 4 (disabling).

### Patient-reported outcomes

European Organisation of Research and Treatment of Cancer-quality of life CIPN questionnaire (EORTC-CIPN20) [9] was used to assess CIPN burden in the past 7 days via 20 questions, each rated on a scale from 1 (not at all) to 4 (very much). The scores were converted to a 0–100 scale, with higher scores indicating more symptom burden. The CIPN-Rasch-built Overall Disability Score (CIPN-R-ODS) [10] was used to assess patient disability. The total score of 56 was converted to a 0–100 scale, with lower scores indicating greater symptom burden.

### Sensory and Functional assessment

Functional ability of the upper-limb was assessed using the Grooved Pegboard Test, a timed task to assess dexterity and fine motor control where patients were required to place 25 keyhole-shaped pegs on a 5 × 5 board with random orientation [11].

Sensory function was assessed on digit 2 of the dominant hand. Briefly, grating orientation threshold was determined using JVP domes (Stoelting Co.) with grates of variable width presented randomly to determine detection threshold in mm [12]. Similarly, mechanical detection threshold in mN was determined using Von-Frey monofilaments (Marstock Nervtest, Germany) [13].

### Neurological assessments

Total Neuropathy Score-clinical version (TNSc, ©Johns Hopkins University) was used to assess neuropathy across 6 domains: patient-reported sensory and motor symptoms; upper and lower-limb pinprick and vibration sensitivity (128 Hz tuning fork); lower-limb strength and deep tendon reflexes [14, 15]. Each domain was graded from 0 to 4, for a total score of 0 to 24, with higher scores indicating greater neuropathy.

Nerve conduction studies were conducted using a Synergy Medelec Electromyograph (Natus Medical Inc.). Maximum compound motor and sensory action potentials from the tibial and sural nerves, respectively, were recorded using standard protocols [16].

### Statistical analysis

Distribution of data was assessed using the Shapiro-Wilk test, with normal data presented as mean ± standard deviation and non-normal data presented as median (interquartile range). Comparisons between groups and across chemotherapies were assessed using Mann–Whitney U test for continuous data and Chi-Square test for categorical data. Statistical significance was achieved when *p* < 0.05. Statistical analyses were performed using SPSS statistical package (v26) and GraphPad Prism 10.

## Results

### Chronic peripheral neuropathy following nab-paclitaxel treatment

A cohort of 24 patients were assessed following completion of nab-paclitaxel treatment, at a median of 3.5 (interquartile range (IQR) 10) months since treatment (Table 1). In total, 33% (*n* = 8) experienced a dose reduction or premature cessation of nab-paclitaxel treatment due to CIPN. At the time of assessment, 71% (*n* = 17) of patients reported persistent CIPN, which was mild (National Cancer Institute Sensory Neuropathy Subscale grade 1) in 10 patients and moderate/severe in 7 patients. Overall, 16 patients (67%) were above the EORTC-CIPN20 threshold for minimally significant CIPN, and 7 (29%) were above the threshold for clinically significant CIPN [17].

**Table 1:** Cohort clinical characteristics.

Characteristics	Nab-paclitaxel *n* = 24	Paclitaxel *n* = 24	Oxaliplatin *n* = 24	*p*-value^a^ (paclitaxel/oxaliplatin)
Age at trial entry (years) Median (IQR)	64.5 (13)	63.5 (15)	65 (11)	0.82/0.52
Sex- male/female (*n*)	7/17	1/23	14/10	0.02/0.04
BMI (mean ± SD)	25.1 ± 4.8	25.4 ± 4.7	26.1 ± 3.9	0.97/0.39
Diabetes (*n*)	7	7	6	0.50/0.63
Cancer type (*n*)				
Pancreatic	16	-	1	-
Colorectal	-	-	21	-
Breast	7	14	-	-
Gynecological	1	8	-	-
Other (gastrointestinal, esophageal)	-	2	2	-
Cancer stage (*n*)				
I – III	8	22	16	-
IV	16	2	8	-
Total dose (mg/m^2^) Median (IQR)	1,495 (744)	960 (250)	831 (258)	-
Relative dose intensity	-	0.92 (0.17)	0.81 (0.23)	-
Neurotoxic therapy duration (weeks)	22.5 (20.9)	11 (7.1)	22.1 (4.8)	< 0.0001/0.51
Time since treatment (months) Median (IQR)	3.5 (10)	3.5 (12)	3.5 (8)	0.97/0.97
Treatment regimens	Nab-paclitaxel (125 mg/m^2^) on days 1, 8, and 15 with gemcitabine every 28 days (*n* = 16)	Weekly paclitaxel (80 mg/m^2^; *n* = 14); weekly paclitaxel (50 mg/m^2^) and carboplatin (*n* = 1)	2-Weekly FOLFOX (*n* = 20)	
	Nab-paclitaxel (100 mg/m^2^) on days 1, 8, and 15 (*n* = 7)	3-Weekly paclitaxel (175 mg/m^2^) and carboplatin (*n* = 6)	2-Weekly FOLFIRINOX (*n* = 1)	
	Nab-paclitaxel (150 mg/m^2^) weekly (*n* = 1)	Paclitaxel (80 mg/m^2^) on day 1, 8, 15 and 3-weekly carboplatin (*n* = 3)	3-weekly XELOX (*n* = 3)	

Cross-sectionally recruited cohort demographics. BMI: body mass index; SD: standard deviation; IQR: interquartile range

a Statistical significance comparing nab-paclitaxel group to paclitaxel group/nab-paclitaxel group to oxaliplatin group.

Numbness was the most frequently reported symptom, with 63% reporting numbness (*n* = 15), 38% reporting tingling (*n* = 9), and 21% reporting burning or shooting pain (*n* = 5).

Functional difficulties were commonly reported (42%, *n* = 10), including 25% reporting problems walking (*n* = 6), 17% reporting difficulties doing up buttons (*n* = 4), and 21% reporting difficulty holding a pen (*n* = 5). 54% (*n* = 13) reported difficulty opening a jar or bottle due to hand weakness. Lower-limb symptoms were reported by 58% of patients (*n* = 14), with upper-limb symptoms noted in 33% (*n* = 8).

Patients were separated into two groups based on time post-treatment: 0–4 months post-treatment (*n* = 13; 0 (3) months post-treatment) and ≥ 6 months post-treatment (*n* = 11; 11 (20) months; Supplementary Tables 1 and 2). There were no differences in age, body mass index (BMI), or cumulative dose between groups. There were no differences in the majority of CIPN outcomes between groups, but the TNSc score was lower in the > 6 months post-nab-paclitaxel group (*p* = 0.044), and the tibial amplitude was increased (*p* = 0.035). However, there were no differences in patient-reported CIPN (*p* = 0.331), patient-reported disability (*p* = 0.865), or sural amplitude (*p* = 0.426).

### Comparison with oxaliplatin and paclitaxel-treated cohorts

Nab-paclitaxel-treated patients were compared to matched cohorts of oxaliplatin (*n* = 24) and paclitaxel-treated patients (*n* = 24) (Table 1; Supplementary Table 3). There were no significant differences in age, BMI, or time since treatment between cohorts. In total, 13 oxaliplatin-treated patients (54%) and 8 paclitaxel-treated patients (33%) experienced a dose reduction or early cessation of paclitaxel due to neuropathy.

**Figure 1:**
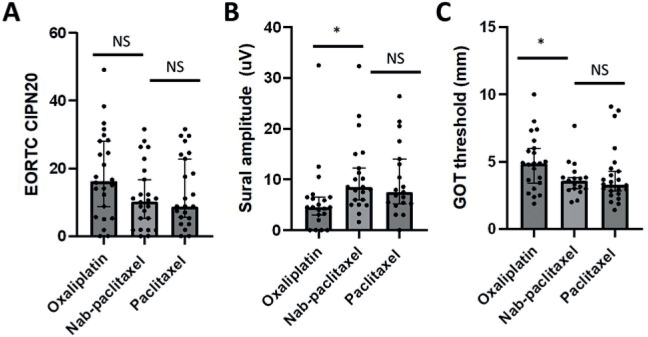
Comparison of neuropathy severity between cohorts. Data points for oxaliplatin (left), nab-paclitaxel (middle), and paclitaxel (right) cohorts for (A) patient-reported neuropathy (EORTC-CIPN20 questionnaire; range 0–100, higher values corresponding to greater neuropathy), (B) sural amplitude (values in μV, lower values corresponding to greater neuropathy), and (C) grating orientation threshold (GOT; values in mm, higher values corresponding to greater neuropathy). Bars indicate median with error bars indicating 95% CI. Statistical comparisons between nab-paclitaxel and other cohorts are depicted, with NS indicating not significant and * indicating *p* < 0.05.

Overall, there were no differences in any CIPN severity measure between nab-paclitaxel and paclitaxel-treated patients (Table 2; Figure 1). Similarly, there were no differences in any parameter between the nab-paclitaxel and paclitaxel groups (*n* = 13 each) between 0–4 months and > 6 months post-treatment groups (*n* = 11 each; Supplementary Tables 1 and 2).

**Table 2:** Neuropathy characteristics.

Median (IQR)	Nab-paclitaxel *n* = 24	Paclitaxel *n* = 24	Oxaliplatin *n* = 24	*p*-value^a^ (paclitaxel/oxaliplatin)
TNSc^b^	4.0 (4)	4.0 (5)	5.0 (4)	0.51/0.12
Sural amplitude^c^ (µV)	8.5 (8)	7.5 (9)	4.5 (4)	0.65/0.003
Tibial amplitude^c^ (mV)	7.2 (7)	8.2 (7)	7.7 (4)	0.81/0.97
NCI Grade 0	7 (29%)	4 (17%)	4 (17%)	-
NCI Grade 1	10 (42%)	6 (25%)	9 (37%)	-
NCI Grade 2/3	7 (29%)	14 (58%)	11 (46%)	-
Patient-reported outcomes				
EORTC-QLQ-CIPN20^b^	11.7 (16)	8.8 (19)	16.2 (22)	0.66/0.06
R-ODS CIPN^c^	90.0 (21)	84.0 (19)	85.5 (23)	0.44/0.73
Sensory discrimination tasks				
Grooved pegboard^b^ (s)	78.9 (33)	67.9 (22)	81.2 (30)	0.35/0.70
Grating orientation task^b^ (mm)	3.6 (1)	3.3 (2)	4.9 (3)	0.77/0.042
Von-Frey monofilaments^b^ (mN)	0.18 (0.2)	0.23 (0.7)	0.71 (2.6)	0.40/0.006

a Statistical significance comparing nab-paclitaxel group to paclitaxel group/nab-paclitaxel group to oxaliplatin group;

b Higher score is associated with greater neuropathy;

c Lower score is associated with greater neuropathy.

NCI: National Cancer Institute Sensory Neuropathy Subscale; IQR: interquartile range.

Oxaliplatin-treated patients demonstrated reduced sural amplitude (*p* = 0.003) and increased grating orientation (*p* = 0.042) and Von Frey sensory thresholds compared to nab-paclitaxel treated patients (*p* = 0.006), highlighting a greater neuropathy burden (Table 2; Figure 1). However, there were no differences in patient-reported CIPN severity or neurologically graded CIPN severity between nab-paclitaxel and oxaliplatin cohorts (Table 2). When examining the 0–4 months post-treatment groups, it was observed that there were differences in sensory thresholds between nab-paclitaxel and oxaliplatin cohorts (*n* = 13 each; Supplementary Table 1). In the > 6 months post-treatment groups, oxaliplatin-treated patients (*n* = 11) demonstrated reduced sural amplitude (*p* < 0.001) and elevated Von Frey monofilament threshold (*p* = 0.042), as well as greater TNSc (*p* = 0.028) (Supplementary Table 2).

## Discussion

CIPN is a significant complication of chemotherapy treatment associated with reduced treatment tolerability, producing functional deficits and reduced quality of life. There have been limited studies examining the natural history of nab-paclitaxel-induced CIPN outcomes. This study used neurological assessment tools combining objective clinical examination, functional testing, neurophysiological assessment, and patient-reported outcomes to investigate post-treatment outcomes.

Nab-paclitaxel produced chronic CIPN in the majority of patients (71%), although the majority of cases were mild. Despite this, 42% of patients reported that CIPN affected their function in some capacity. Importantly, 33% of patients experienced a dose modification of nab-paclitaxel treatment due to CIPN, which underscores the clinical relevance of accurately assessing CIPN. Neurologically graded CIPN severity was improved in the cohort who had a longer time elapsed since nab-paclitaxel treatment, highlighting the potential for recovery in some patients.

These findings are consistent with prior work, with a meta-analysis of CIPN in nab-paclitaxel trials determining an overall incidence of 51% [7], while 68% of nab-paclitaxel-treated patients reported chronic CIPN at 1-year post-treatment [18]. However, while a neurotoxicity analysis of nab-paclitaxel/gemcitabine therapy in pancreatic cancer patients found that 54% of patients developed CIPN [5], the median resolution time for severe CIPN was 1 month. This is not consistent with the CIPN profile presented in this study, which found that substantial chronic CIPN persisted post-treatment beyond 1 month. These differences may relate to the method of CIPN assessment, as clinician-reported neuropathy metrics are less sensitive compared to other assessment tools [19].

Previous studies have failed to reach a conclusion on the relative neurotoxicity of nab-paclitaxel compared to paclitaxel. Some clinical studies have suggested reduced patient and clinician-reported neuropathy severity with nab-paclitaxel [20], while a meta-analysis of nab-paclitaxel trials found no difference in risk of CIPN between nab-paclitaxel and paclitaxel [7]. Similarly, a prospective study of 58 breast cancer patients revealed no differences in patient-reported CIPN severity compared to paclitaxel-treated patients [18]. In contrast, other meta-analyses concluded that nab-paclitaxel was associated with higher incidence of CIPN than paclitaxel [6, 21]. Similarly, a study of 295 nab-paclitaxel-treated breast cancer patients found greater patient-reported sensory CIPN compared to other taxanes after 6 weeks [22].

The present study did not find any differences in the severity or incidence of neuropathy between nab-paclitaxel and paclitaxel-treated patients. This is different to the results of some prior work. However, the majority of prior studies have used a limited range of assessment tools, including clinician-reported outcomes, which may be less sensitive than other tools in identifying CIPN [23]. By utilizing a range of assessment tools, including patient-reported outcomes and neurological grading scales, this study provides more evidence to compare the neuropathy burden in nab-paclitaxel and paclitaxel-treated patients. The use of multimodal assessment tools is recommended in research settings to examine CIPN and its impacts across multiple domains and to identify differences in neuropathy burden between cohorts [24]. However, in the clinical setting, routine assessment with a variety of assessment tools is typically not practical. Of note, patient-reported outcomes have been demonstrated to be valid and helpful to assist in delineating neuropathy burden in the clinical setting [25].

Other factors that may contribute to the differences with prior analyses include demographic and cohort differences. There has been a limited analysis in prior meta-analyses of potential covariates that may affect neuropathy risk [26, 27]. Age and BMI have been identified as risk factors for nab-paclitaxel CIPN [28, 29], suggesting that it may be important to control for these risk factors in future studies. As the comparison cohorts in our study were matched for age and BMI, the impact of inter-group differences may have been mitigated. Finally, there are additional risk factors, including genetic factors [30], which may govern CIPN development and lead to differences in CIPN incidence across cohorts.

In comparison to nab-paclitaxel, oxaliplatin was associated with more severe neuropathy. In particular, > 6 months post-treatment, there was significantly greater neurologically graded neuropathy and reduced sensory nerve amplitude, as well as elevated sensory thresholds compared to nab-paclitaxel-treated patients. This is in-line with the ‘coasting’ phenomenon that is reported following oxaliplatin treatment [31], whereby CIPN can worsen following treatment. Although neuropathy burden was greater in oxaliplatin-treated patients, we did not identify any difference in patient-reported symptoms or disability. This may be due to the low sample size of the present study or may also reflect differences in how the impact of nerve damage is rated by patients.

This study was a cross-sectional analysis of patients, limited by a low sample size. Analysis of the longitudinal development of CIPN during nab-paclitaxel treatment in comparison to other chemotherapies would provide further insight into the time course, progression, and recovery of CIPN. However, nab-paclitaxel is often utilized in patient groups with advanced disease, which limits the ability to follow patients long-term, due to high potential for study drop-out. The present sample is likely representative of patients who were well enough to complete the study following their treatment, and it is possible that patients with greater disease burden may have a different toxicity profile. Finally, we included a heterogenous group of patients with different cancer types (nab-paclitaxel treatment for either pancreatic or breast cancer, paclitaxel treatment for breast or ovarian cancer), who may have experienced different CIPN outcomes due to demographic, cancer type, or regimen differences.

While the patients were matched across multiple variables, nab-paclitaxel, paclitaxel, and oxaliplatin are typically used for different cancer types, different cancer stages, and in combination with other chemotherapies, and this may impact on toxicity outcomes. Nab-paclitaxel is often used in the metastatic cancer setting, following previous lines of therapy. Accordingly, there were more stage IV patients in the nab-paclitaxel-treated group. Prior therapy has been associated with greater risk of CIPN with subsequent chemotherapy [32]. Although multiple studies have not identified an impact of cancer stage on CIPN [33, 34], some analyses have identified higher risk with lower cancer stages [35]. Advanced disease stage may be associated with functional decline and other comorbidities such as risk of falls [36], which may influence neuropathy assessments.

This study demonstrates that nab-paclitaxel is associated with similar chronic CIPN compared to paclitaxel, but oxaliplatin produced more severe chronic neuropathy. Understanding agent-specific CIPN profiles and the potential factors that influence neuropathy risk are important to enable clinicians to monitor at-risk patients during treatment. Appropriate identification of CIPN via routine surveillance will enable prompt consideration of dose-modification and referral to appropriate services [37] to improve patient care and quality of life.

## Supplementary Material

Comparison of nab-paclitaxel, paclitaxel, and oxaliplatin-induced peripheral neuro-pathy: a cross-sectional cohort study

## Data Availability

Data are available from the authors upon reasonable request.

## References

[1] Park SB, Goldstein D, Krishnan AV, Lin CS-Y, Friedlander ML, Cassidy J, et al. Chemotherapy-induced peripheral neurotoxicity: a critical analysis. CA Cancer J Clin. 2013;63:419–37. 10.3322/caac.21204 24590861

[2] Hertz DL, Childs DS, Park SB, Faithful S, Ke Y, Ali NT, et al. Patient-centric decision framework for treatment alterations in patients with chemotherapy-induced peripheral neuropathy (CIPN). Cancer Treat Rev. 2021;99:102241. 10.1016/j.ctrv.2021.102241 34174668

[3] Pachman DR, Qin R, Seisler D, Smith EML, Kaggal S, Novotny P, et al. Comparison of oxaliplatin and paclitaxel-induced neuropathy (Alliance A151505). Support Care Cancer. 2016;24:5059–68. 10.1007/s00520-016-3373-1 27534963 PMC5439148

[4] Timmins HC, Li T, Trinh T, Kiernan MC, Harrison M, Boyle F, et al. Weekly paclitaxel-induced neurotoxicity in breast cancer: outcomes and dose response. Oncologist. 2021;26(5):366–74. 10.1002/onco.13697 33523545 PMC8100541

[5] Goldstein D, Von Hoff DD, Moore M, Greeno E, Tortora G, Ramanathan RK, et al. Development of peripheral neuropathy and its association with survival during treatment with nab-paclitaxel plus gemcitabine for patients with metastatic adenocarcinoma of the pancreas: a subset analysis from a randomised phase III trial (MPACT). Eur J Cancer. 2016;52:85–91. 10.1016/j.ejca.2015.10.017 26655559

[6] Guo X, Sun H, Dong J, Feng Y, Li H, Zhuang R, et al. Does nab-paclitaxel have a higher incidence of peripheral neuropathy than solvent-based paclitaxel? Evidence from a systematic review and meta-analysis. Crit Rev Oncol Hematol. 2019;139:16–23. 10.1016/j.critrevonc.2019.04.021 31112878

[7] Peng L, Bu Z, Ye X, Zhou Y, Zhao Q. Incidence and risk of peripheral neuropathy with nab-paclitaxel in patients with cancer: a meta-analysis. Eur J Cancer Care (Engl). 2017;26(5):12407. 10.1111/ecc.12407 26537178

[8] Timmins HC, Li T, Huynh W, Kiernan MC, Baron-Hay S, Boyle F, et al. Electrophysiological and phenotypic profiles of taxane-induced neuropathy. Clin Neurophysiol. 2020;131(8):1979–85. 10.1016/j.clinph.2020.02.028 32291143

[9] Postma TJ, Aaronson NK, Heimans JJ, Muller MJ, Hildebrand JG, Delattre JY, et al. The development of an EORTC quality of life questionnaire to assess chemotherapy-induced peripheral neuropathy: the QLQ-CIPN20. Eur J Cancer. 2005;41:1135–9. 10.1016/j.ejca.2005.02.012 15911236

[10] Binda D, Vanhoutte EK, Cavaletti G, Cornblath DR, Postma TJ, Frigeni B, et al. Rasch-built overall disability scale for patients with chemotherapy-induced peripheral neuropathy (CIPN-R-ODS). Eur J Cancer. 2013;49(13):2910–18. 10.1016/j.ejca.2013.04.004 23668917

[11] Schmidt SL, Oliveira RM, Rocha FR, Abreu-Villaca Y. Influences of handedness and gender on the grooved pegboard test. Brain Cognit. 2000;44:445–54. 10.1006/brcg.1999.1204 11104536

[12] Craig JC. Grating orientation as a measure of tactile spatial acuity. Somatosens Mot Res. 1999;16:197–206. 10.1080/08990229970456 10527368

[13] Rolke R, Baron R, Maier C, Tölle TR, Treede DR, Beyer A, et al. Quantitative sensory testing in the German Research Network on Neuropathic Pain (DFNS): standardized protocol and reference values. Pain. 2006;123(3):231–43. 10.1016/j.pain.2006.01.041 16697110

[14] Cavaletti G, Jann S, Pace A, Plasmati R, Siciliano G, Briani C, et al. Multi-center assessment of the total neuropathy score for chemotherapy-induced peripheral neurotoxicity. J Peripher Nerv Syst. 2006;11:135–41. 10.1111/j.1085-9489.2006.00078.x 16787511

[15] Cornblath DR, Chaudhry V, Carter K, Lee D, Seysedadr M, Miernicki M, et al. Total neuropathy score: validation and reliability study. Neurology. 1999;53:1660. 10.1212/WNL.53.8.1660 10563609

[16] Liveson JA, Ma DM. Laboratory reference for clinical neurophysiology. Oxford University Press, New York; 1992.

[17] Li T, Timmins HC, Trinh T, Mizrahi D, Harrison M, Horvath LG, et al. Patient-reported outcome measures in chemotherapy-induced peripheral neurotoxicity: defining minimal and clinically important changes. J Natl Compr Canc Netw. 2023;21(2):125–32.e3. 10.6004/jnccn.2022.7074 36791763

[18] Kida K, Yamada A, Shimada K, Narui K, Sugae S, Shimizu D, et al. A prospective comparison study utilizing patient-reported outcomes of taxane-related peripheral neuropathy between nab-paclitaxel and standard paclitaxel in patients with breast cancer. Breast Cancer. 2024;31(3):409–16. 10.1007/s12282-024-01551-z 38453739

[19] Tan A, McCrary JM, Park SB, Trinh T, Goldstein D. Chemotherapy-induced peripheral neuropathy-patient-reported outcomes compared with NCI-CTCAE grade. Support Care Cancer. 2019;27(12):4771–7. 10.1007/s00520-019-04781-6 30972648

[20] Hirsh V, Okamoto I, Hon JK, Page RD, Orsini J, Sakai H, et al. Patient-reported neuropathy and taxane-associated symptoms in a phase 3 trial of nab-paclitaxel plus carboplatin versus solvent-based paclitaxel plus carboplatin for advanced non-small-cell lung cancer. J Thorac Oncol. 2014;9:83–90. 10.1097/JTO.0000000000000011 24346096

[21] Deng X, Huang X, Dong X, Mao G, Xing W. Efficacy and safety of nanopaclitaxel formulation for cancer treatment: evidence from randomized clinical trials. Nanomedicine. 2023;18(10):833–43. 10.2217/nnm-2023-0080 37222128

[22] Mo H, Yan X, Zhao F, Teng Y, Sun X, Lv Z, et al. Association of taxane type with patient-reported chemotherapy-induced peripheral neuropathy among patients with breast cancer. JAMA Netw Open. 2022;5(11):e2239788. 10.1001/jamanetworkopen.2022.39788 36322088 PMC9631104

[23] McCrary JM, Goldstein D, Trinh T, Timmins HC, Li T, Friedlander M, et al. Optimizing clinical screening for chemotherapy-induced peripheral neuropathy. J Pain Sympt Manag. 2019;58:1023–32. 10.1016/j.jpainsymman.2019.07.021 31374367

[24] Alberti P, Rossi E, Cornblath DR, Merkies ISJ, Postma TJ, Frigeni B, et al. Physician-assessed and patient-reported outcome measures in chemotherapy-induced sensory peripheral neurotoxicity: two sides of the same coin. Ann Oncol. 2014;25(1):257–64. 10.1093/annonc/mdt409 24256846 PMC3868322

[25] Li T, Timmins HC, Mahfouz FM, Trinh T, Mizrahi D, Horvath LG, et al. Validity of patient-reported outcome measures in evaluation nerve damage following chemotherapy. JAMA Netw Open. 2024;7(8):e2424139. 10.1001/jamanetworkopen.2024.24139 39120903 PMC11316238

[26] Timmins HC, Li T, Goldstein D, Trinh T, Mizrahi D, Harrison M, et al. The impact of obesity on neuropathy outcomes for paclitaxel- and oxaliplatin-treated cancer survivors. J Cancer Surviv. 2022;16(2):223–32. 10.1007/s11764-021-01012-y 33641031

[27] Mizrahi D, Park SB, Li T, Timmins HC, Trinh T, Au K, et al. Hemoglobin, body mass index, and age as risk factors for paclitaxel- and oxaliplatin-induced peripheral neuropathy. JAMA Netw Open. 2021;4(2):e2036695. 10.1001/jamanetworkopen.2020.36695 33587134 PMC7885037

[28] Catalano M, Ramello M, Conca R, Aprile G, Petrioli R, Roviello G. Risk factors for nab-paclitaxel and gemcitabine-induced peripheral neuropathy in patients with pancreatic cancer. Oncology. 2022;100(7):384–91. 10.1159/000524868 35551139

[29] Kanbayashi Y, Sakaguchi K, Ishikawa T, Tabuchi Y, Takagi R, Yokota I, et al. Predictors of the development of nab-paclitaxel-induced peripheral neuropathy in breast cancer patients: post hoc analysis of a prospective, phase II, self-controlled clinical trial. Med Oncol. 2022;39(10):153. 10.1007/s12032-022-01754-4 35852641

[30] Hooshmand K, Goldstein D, Timmins HC, Li T, Harrison M, Friedlander ML, et al. Polygenic risk of paclitaxel-induced peripheral neuropathy: a genome-wide association study. J Transl Med. 2022;20(1):564. 10.1186/s12967-022-03754-4 36474270 PMC9724416

[31] Argyriou AA, Polychronopoulos P, Iconomou G, Chroni E, Kalofonos HP. A review on oxaliplatin-induced peripheral nerve damage. Cancer Treat Rev. 2008;34:368–77. 10.1016/j.ctrv.2008.01.003 18281158

[32] Battaglini E, Goldstein D, Grimison P, MuCullough S, Mendoza-Jones P, Park SB. Chemotherapy-induced peripheral neurotoxicity in cancer survivors: predictors of long-term patient outcomes. J Natl Compr Canc Netw. 2021;19(7):821–8. 10.6004/jnccn.2021.7026 34340206

[33] Hershman DL, Till C, Wright JD, Awad D, Ramsey SD, Barlow WE, et al. Comorbidities and risk of chemotherapy-induced peripheral neuropathy among participants 65 years or older in Southwest Oncology Group clinical trials. J Clin Oncol. 2016;34(25):3014–22. 10.1200/JCO.2015.66.2346 27325863 PMC5012713

[34] Molassiotis A, Cheng HL, Leung KT, Li YC, Wong KH, Au JSK, et al. Risk factors for chemotherapy-induced peripheral neuropathy in patients receiving taxane- and platinum-based chemotherapy. Brain Behav. 2019;9(6):e01312. 10.1002/brb3.1312 31063261 PMC6576180

[35] Greenwald MK, Ruterbusch JJ, Beebe-Dimmer JL, Simon MS, Albrecht TL, Schwartz AG. Risk of incident claims for chemotherapy-induced peripheral neuropathy among women with breast cancer in a Medicare population. J Am Canc Soc. 2019;125(2):269–77. 10.1002/cncr.31798 PMC6329662 30387871

[36] Hines RB, Schoborg C, Sumner T, Thiesfeldt D, Zhang S. The associations of oxaliplatin-induced peripheral neuropathy, sociodemographic characteristics, and clinical characteristics with time to fall in older adults with colorectal cancer. Am J Epidemiol. 2024;193(9):1271–80. 10.1093/aje/kwae067 38751324 PMC11483325

[37] Mizrahi D, Goldstein D, Kiernan MC, Robinson L, Pitiyarachchi O, McCullough S, et al. Development and consensus process for a clinical pathway for the assessment and management of chemotherapy-induced peripheral neuropathy. Support Care Cancer. 2022;30:5965–74. 10.1007/s00520-022-07024-3 35394563 PMC9135801

