# Supplementary material for: Comparison of nab-paclitaxel, paclitaxel, and oxaliplatin-induced peripheral neuro-pathy: a cross-sectional cohort study
Source: Acta Oncol. 2025 Apr 15;64:42935. doi: 10.2340/1651-226X.2025.42935 (PMC12012729; doi:10.2340/1651-226X.2025.42935)
Supplement: Comparison of nab-paclitaxel, paclitaxel, and oxaliplatin-induced peripheral neuro-pathy: a cross-sectional cohort study [file AO-64-42935-s1.pdf]

Supplementary material has been published as submitted. It has not been copyedited, or typeset by Acta Oncologica

Supplementary table 1. Neuropathy characteristics 0-4 months post treatment

Median (IQR)	Nab-paclitaxel n=13	Paclitaxel n=13	Oxaliplatin n=13	p-value[a] (Paclitaxel / Oxaliplatin)
TNSc[b]	4.5 (4)	4 (6)	5 (5)	0.94/1.00
Sural amplitude[c] (μV)	8.2 (7.8)	6.7 (6.6)	5.9 (10.5)	0.67/0.25
Tibial amplitude[c] (mV)	5.8 (7.2)	7.7 (6.4)	7.4 (6.3)	0.42/0.15
NCI Grade 0	3 (23%)	3 (23%)	2 (15.5%)	--
NCI Grade 1	7 (54%)	3 (23%)	2 (15.5%)	
NCI Grade 2/3	3 (23%)	7 (54%)	9 (69%)	
Patient reported outcomes				
EORTC-QLQ-CIPN20[b]	8.8 (8.7)	18.5 (23.4)	15 (27)	0.20/0.08
R-ODS CIPN[c]	90 (23)	80 (15)	90 (21)	0.24/0.92
Sensory and Functional assessments				
Grooved pegboard[b] (s)	81.4 (30.3)	69.3 (22.5)	82.2 (29.8)	0.39/0.69
Grating orientation task[b] (mm)	3.6 (0.83)	3.3 (2.1)	4.88 (3.1)	0.46/**0.007**
Von-Frey monofilaments[b] (mN)	0.18 (0.41)	0.71 (1.4)	0.71 (6.9)	0.54/0.46
Clinical characteristics				
Time since treatment (months) Median (IQR)	2 (3)	0 (4)	0 (3)	0.90/0.64

Table Note: a. Statistical significance comparing nab-paclitaxel group to paclitaxel group/nab-paclitaxel group to oxaliplatin group.
b. Higher score is associated with greater neuropathy.
c. Lower score is associated with greater neuropathy.

NCI= National Cancer Institute Sensory Neuropathy Subscale

Supplementary Table 2. Neuropathy characteristics >6 months post treatment

Median (IQR)	Nab-paclitaxel n=11	Paclitaxel n=11	Oxaliplatin n=11	p-value[a] (Paclitaxel / Oxaliplatin)
TNSc[b]	2 (5)	4 (4)	5 (3)	0.40/**0.028**
Sural amplitude[c] (μV)	10 (9)	10.4 (18.4)	4.4 (2.2)	1.00/**0.001**
Tibial amplitude[c] (mV)	10.4 (10.2)	10.4 (7.1)	8.0 (4.9)	1.00/0.16
NCI Grade 0	4 (36.5%)	1 (9%)	2 (18%)	--
NCI Grade 1	3 (27%)	6 (54.5%)	4 (36.5%)	
NCI Grade 2/3	4 (36.5%)	4 (36.5%)	5 (45.5%)	
Patient reported outcomes				
EORTC-QLQ-CIPN20[b]	11.1 (24.6)	7.9 (8.8)	16.7 (15.8)	0.35/0.75
R-ODS CIPN[c]	90 (21)	90 (18)	82 (24)	0.85/0.59
Sensory and Functional assessments				
Grooved pegboard[b] (s)	72.4 (44.3)	66.4 (17.9)	72.5 (24.7)	0.81/0.71
Grating orientation task[b] (mm)	3.4 (3.5)	3.3 (1.6)	4.2 (3.2)	0.96/0.71
Von-Frey monofilaments[b] (mN)	0.16 (0.26)	0.18 (.09)	0.66 (1.2)	0.53/**0.042**
Clinical characteristics				
Time since treatment (months) Median (IQR)	11 (20)	13 (17)	9 (24)	0.93/0.66

Table Note: a. Statistical significance comparing nab-paclitaxel group to paclitaxel group/nab-paclitaxel group to oxaliplatin group.
b. Higher score is associated with greater neuropathy.
c. Lower score is associated with greater neuropathy.

NCI= National Cancer Institute Sensory Neuropathy Subscale

Supplementary Table 3. Neuropathy characteristics

	**Nab-paclitaxel (n=24)**	**Paclitaxel (n=24)**	**Oxaliplatin (n=24)**
**Numbness**	15 (63%)	12 (50%)	21 (88%)
**Tingling**	9 (38%)	16 (67%)	19 (79%)
**Burning or shooting pain**	5 (21%)	7 (29%)	4 (17%)
**Problems walking**	6 (25%)	9 (38%)	10 (42%)
**Difficulties doing up buttons**	4 (17%)	8 (33%)	14 (58%)
**Difficulty holding a pen**	5 (21%)	4 (17%)	9 (38%)
**Difficulty opening a jar or bottle due to weakness**	13 (54%)	13 (54%)	11 (46%)
**Lower-limb symptoms**	14 (58%)	18 (75%)	22 (92%)
**Upper-limb symptoms**	8 (33%)	13 (54%)	18 (75%)

Table Note: Neuropathy characteristics derived from the EORTC-CIPN20 questionnaire. Numbers reported who indicated a response of ‘a little bit’, ‘quite a bit’ or ‘very much’ or above for numbness (question 3 or 4), tingling (question 1 or 2), burning or shooting pain (question 5 or 6), problems walking (question 9), difficulties doing up buttons (question 12), difficulty holding a pen (11), difficulty opening a jar or bottle due to weakness (question 13), lower limb symptoms (question 2 or 4), and upper limb symptoms (question 1 or 3).
